# Functional Analysis of Tomato SPDS in Response to Osmotic Stress

**DOI:** 10.3390/cells15060533

**Published:** 2026-03-17

**Authors:** Lilan Cheng, Jingling Zhang, Chenyu Lin, Wenjuan Wang, Siyuan Huang, Liyun Yang, Jie Li, Xin Guo, Xiaohui Yu

**Affiliations:** School of Tropical Agriculture and Forestry, Hainan University, Haikou 570228, China; chenglilan@hainanu.edu.cn (L.C.); jeanz@hainanu.edu.cn (J.Z.); chenyulin@hainanu.edu.cn (C.L.); lupengyu@yzwlab.cn (W.W.); 21110901000011@hainanu.edu.cn (S.H.); 996674@hainanu.edu.cn (L.Y.); 22220951310216@hainanu.edu.cn (J.L.)

**Keywords:** *Solanum lycopersicum*, spermidine synthase (SPDS), polyamine metabolism, abiotic stress, gene expression, fruit development

## Abstract

**Highlights:**

**What are the main findings?**

- Four *SlSPDS* genes were identified in tomato, and their characteristics, subcellular localization, and stress response patterns were systematically analyzed.
- Transient expression of *SlSPDS* affected polyamine contents in tomato, and overexpression transgenic tomato lines were generated to verify the function of *SlSPDS*.

**What is the implication of the main finding?**

- These findings clarify the potential role of the *SlSPDS* gene family in abiotic stress tolerance in tomato.
- This study provides a theoretical basis for the genetic improvement of stress resistance in Solanaceae crops.

**Abstract:**

Polyamines, such as spermidine (Spd), are small aliphatic amines that play critical roles in plant growth, fruit development, and stress responses. Spermidine synthase (SPDS) is the enzyme responsible for catalyzing Spd biosynthesis. However, the functional characterization of *SPDS* genes in tomato (*Solanum lycopersicum*) has been less studied. In this study, four *SlSPDS* genes (*SlSPDS1-4*) were identified and analyzed for their physicochemical properties, phylogenetic relationships, promoter cis-acting elements, subcellular localization, responses to various abiotic stresses, and effects on polyamine content in tomato leaves. Promoter analysis revealed the presence of multiple hormone and stress-responsive elements. Simultaneously, the overexpressing lines were subjected to osmotic stress treatment. Subcellular localization experiments demonstrated that SlSPDS1 and SlSPDS2 were distributed in both the nucleus and cytoplasm, while SlSPDS3 and SlSPDS4 were specifically localized to the nucleus. *SlSPDS1-3* exhibited significant responses to high/low temperature stress, salt stress, and ABA stress. Meanwhile, only *SlSPDS1* and *SlSPDS4* exhibited responses to drought stress. Transient expression of *SlSPDSs* in tomato revealed changes in the accumulation levels of spermine, putrescine, tyramine, and tryptamine, whereas the contents of spermidine and phenethylamine showed no significant changes. Simultaneously, we successfully obtained four *SlSPDS*-overexpressing transgenic tomato lines, OE-*SPDS1-4*. Phenotypic analysis confirmed that these transgenic lines exhibited significantly reduced wilting and chlorosis compared with WT plants under drought and salt stress. Functional validation indicates that overexpression of these genes enhances reactive oxygen species (ROS) scavenging capacity in transgenic tomatoes, thereby potentially improving their tolerance to drought and salt stress. These findings highlighted the potential function of *SlSPDS* genes in tomato, providing valuable targets for improving stress tolerance.

## 1. Introduction

Polyamines (PAs) are a class of aliphatic nitrogenous bases widely distributed in living organisms, playing a central role in plant growth, development, and stress adaptation [1]. In plants, polyamines primarily comprise three major categories: putrescine (Put), spermidine (Spd), and spermine (Spm). Polyamines play crucial biological roles in both animals and plants. Polyamines regulate growth and development, stabilize biomolecules, promote gene transcription and protein synthesis, enhance stress resistance, and modulate aging. In plants, PAs also confer enhanced stress resistance, and Put, in particular, acts as a fast-acting switch to rapidly improve tolerance against osmotic stress [2]. As a key intermediate in the synthesis of putrescine and spermine, spermidine possesses unique and irreplaceable functions. In animals, polyamines regulate aging and neuroprotection via autophagy activation. Singh et al. reported that Spd, a caloric restriction mimetic, protects the aged male rat brain against oxidative stress and apoptosis by activating autophagy [3]. Similarly, Xu et al. reported that Spd and Spm delay brain aging and improve cognitive function in mice via the same autophagic pathway [4]. In plants, polyamines regulate cell growth, differentiation, and proliferation; delay aging; and influence embryonic development, morphogenesis, root formation, floral bud differentiation, and fruit growth [1]. For instance, in *Anoectochilus roxburghii*, low concentrations of exogenous Spd induce premature flowering, while high concentrations inhibit flowering and may even cause plant death [5]. In tobacco thin-layer tissue culture, spermidine promotes floral bud differentiation, while its absence results in the formation of vegetative buds only [6]. Research indicates that polyamine metabolism plays a role in parthenocarpy in tomatoes [7]. Exogenous polyamine application to strawberry fruits enhances fruit coloration [8].

Plant polyamine biosynthesis primarily proceeds via the arginine decarboxylation pathway: arginine is decarboxylated by arginine decarboxylase (ADC, EC 4.1.1.19) to form agmatine, which is subsequently converted to citrulline and then to Put [1,9]. Putrescine is further converted to Spd by spermidine synthase (SPDS, EC 2.5.1.16), and Spd can be catalyzed by spermine synthase (SPMS, EC 2.5.1.22) to Spm. As the rate-limiting enzyme in Spd biosynthesis, *SlSPDS*-encoded spermidine synthase directly modulates endogenous Spd levels and is involved in multiple plant physiological processes [10]. In *Arabidopsis thaliana*, *AtSPDS* is a core member of the polyamine biosynthetic gene family, and its encoded protein harbors the key catalytic domain for Put-to-Spd conversion [11]. Exogenous spermidine enhances tomato seedling tolerance to salt–alkali stress by regulating physiological and biochemical indicators [12]. The cross-regulation between polyamine and ethylene signaling pathways significantly influences tomato’s defense response against pathogens [13]. By regulating endogenous Spd synthesis and accumulation, the *SPDS* gene profoundly influences tomato fruit development and fruit quality formation [14]. The normal expression of tomato *SlSPDS* is crucial for floral organ identity and fruit set, and its dysregulation frequently results in abnormal floral development and failed fruit formation [15]. As a key member of the polyamine family, spermidine can also regulate lifespan by inducing autophagy pathways [16]. Upregulation of key genes in polyamine synthesis is the core regulatory mechanism for enhancing endogenous polyamine levels in tomatoes [17].

Abiotic stresses, including salt, drought, and extreme temperatures, readily trigger metabolic responses characterized by reactive oxygen species (ROS) burst in plants, leading to oxidative stress damage [18]. Overexpression of the S-adenosyl-L-methionine synthase gene (SAMS) significantly enhances tomato tolerance to alkaline stress by promoting polyamine biosynthesis and maintaining cellular ion homeostasis [19]. Meanwhile, accumulated polyamines can mediate the NO signaling pathway, enhancing ROS scavenging capacity to mitigate oxidative damage [20,21]. Overexpression of *SPDS* genes or exogenous spermidine treatment significantly improves tomato salt tolerance by maintaining ion homeostasis and enhancing antioxidant defense system activity [22]. This regulatory pattern shares similarities with those involving TOR kinase and ABA receptors [23]. For instance, the SlWRKY81-JAZ1-JA signaling pathway regulates spermidine synthesis, and spermidine synergistically enhances tomato salt and alkalinity tolerance by maintaining sodium–potassium ion balance [24]. The effects of exogenous spermidine on drought stress in barley indicate that exogenous spermidine can alleviate drought stress by enhancing antioxidant enzyme activity and other mechanisms [25]. Polyamine metabolism is a key pathway in plant responses to abiotic stresses like drought [26]. Additionally, Pseudomonas strains can alleviate drought stress by regulating the polyamine biosynthesis pathway in Arabidopsis [27]. Exogenous spermidine significantly improves seed germination rates in peppers under drought stress by enhancing root vitality, boosting antioxidant enzyme activity, and promoting the synthesis of osmoprotectants [28]. Under low-temperature stress, overexpression of the *CaSPDS* gene in peppers exacerbates seedling cold injury and elevates reactive oxygen species levels [29]. Similarly, overexpression of the *SPDS* gene in tomatoes enhances cold tolerance by promoting the accumulation of osmoprotective substances and boosting antioxidant enzyme activity [30]. Exogenous spermidine delays chlorophyll degradation in cucumber leaves under high-temperature stress [31]. Under osmotic stress, putrescine synergistically enhances plant stress resistance [2]; abiotic stress induces polyamine accumulation in plants and elevates the activity of key synthetic enzymes such as ADC, SAMDC, and SPDS [32]. Changes in spermidine content in soybeans affect diamine oxidase activity [33]. In eggplant, the transcription factor *SmMYB44* confers enhanced resistance to bacterial wilt by directly activating *SmSPDS* expression and thereby promoting spermidine accumulation [34]. We investigated the multi-stress response mechanism of the tomato *SlSPDS* gene family and found that its overexpression enhances stress tolerance by boosting ROS scavenging and slowing chlorophyll degradation. In contrast, polyamine metabolism exhibits a network-like division of labor. Transient overexpression of Spd has limited regulatory effects on Spd itself but specifically modulates the synthesis of other polyamines and aromatic amines. This feature distinguishes it from other species and fills a gap in research on functional differentiation within the *SlSPDS* gene family.

In summary, tomato *SlSPDS*, a core regulator of polyamine biosynthesis, promotes spermidine accumulation, which in turn modulates ion homeostasis and enhances stress tolerance under saline–alkaline stress [35]. However, the function of *SlSPDS1-4* and its contribution to spermidine synthesis remain unclear. We analyzed the expression patterns of *SlSPDS1-4* genes under various abiotic stresses and validated their functional roles in modulating plant polyamine content using transient expression technology. Furthermore, we established transgenic tomato lines overexpressing *SlSPDS1-4* for the first time and examined their physiological responses to drought and salt stress. This study provides critical data and a theoretical foundation for deciphering the regulatory mechanisms of polyamine metabolism in tomato and enabling targeted genetic improvement of polyamine composition. It will advance research into the relationship between polyamine regulation and tomato quality.

## 2. Materials and Methods

### 2.1. Plant Materials and Growth Conditions

The plant materials used in this study included tomato cultivar Micro-Tom (accession number ID taxonomy4081), transgenic tomato lines overexpressing the *SlSPDS* genes (OE-*SPDS1-4*), and Bentham’s tobacco (*Nicotiana benthamiana*) (accession number ID taxonomy ID: 4097). The tomato and *tobacco* were provided by the Key Laboratory of Sustainable Utilization of Tropical Biological Resources, Hainan Province. All experiments were conducted at Hainan University (longitude: 110.326842, latitude: 20.056716). All plant materials were propagated using a growing medium composed of a 3:1 (*v*/*v*) mixture of commercial potting soil and vermiculite. Tomatoes were cultivated in a greenhouse under a 16 h/8 h light/dark cycle at 24–26 °C. Seedlings were harvested at the five-leaf-one-heart stage (4 weeks old) for subsequent stress treatments and related experiments. Tobacco seeds were directly sown onto the seedling substrate, and uniformly growing plants were selected for genetic transformation and subcellular localization analysis. The workflow for constructing *SlSPDS* gene overexpression transgenic tomato lines was as follows: Taq DNA polymerase was used to amplify the full-length coding sequences of *SlSPDS1-4* genes. The amplification products were cloned into the pG1300 vector. Following T4 DNA ligase-mediated ligation, the recombinant plasmid was used for genetic transformation, ultimately yielding OE-*SPDS1-4* transgenic lines. The validation results of *SlSPDS* overexpression lines by qRT-PCR are displayed in Supplementary Figure S6.

### 2.2. Identification of the SPDS Gene Family

The tomato genome sequence was downloaded the tomato genome sequence from JGI [36] (https://phytozome-next.jgi.doe.gov, accessed on 13 September 2023). Using TBtools-II [37] software, we performed BLAST searches against the *Arabidopsis SPDS1-3* amino acid sequences in the tomato genome database (*Solanum lycopersicum* ITAG4.0) and removed duplicates. We obtained the sequences of genomic DNA and cDNA of all *SPDS* members in FASTA format from the NCBI database. The exon–intron structure of each gene was predicted using the online tool GSDS 2.0 [38] (https://gsds.gao-lab.org/Gsds_help.php, accessed on 7 October 2025). Conserved motif analysis of the *SPDS* gene family was performed by the online software MEME Suite [39] (https://meme-suite.org/meme/tools/meme, accessed on 7 October 2025). TBtools-II was used to visualize these data (Supplementary Figure S1).

### 2.3. Protein Physicochemical Characterization

The physicochemical properties of the SlSPDS1-4 proteins were analyzed by online tools. The theoretical isoelectric point (pI), relative molecular weight (MW), instability coefficient (II), aliphatic index (AI), and mean hydrophobicity (GRAVY) of the proteins were systematically evaluated using the ProtParam tool [40] (https://web.expasy.org/protparam, accessed on 1 October 2025). The subcellular localization of SlSPDS proteins was predicted using the WOLF PSORT tool [41] (https://wolfpsort.hgc.jp, accessed on 1 October 2025).

### 2.4. Multiple Sequence Alignment and Phylogenetic Analysis of the SlSPDS1-4

We downloaded potato (*Solanum tuberosum*), tomato, and rice (*Oryza sativa* L.) from the JGI website [36]. We obtained the genome and annotation files for eggplant (*Solanum melongena*) from the Solanaceae Database (https://solgenomics.net/, accessed on 13 October 2025). Additionally, we acquired the protein sequences of SPDS gene family members from *Arabidopsis thaliana* via the TAIR website (https://www.arabidopsis.org, accessed on 13 October 2025). Protein sequences were submitted to InterPro via TBtools-II [37] for domain prediction. Candidate sequences were identified through BLAST alignment using Arabidopsis SPDS protein sequences, with initial screening completed by NCBI BLAST alignment. Tomato SPDS family members were validated using TBtools’ built-in TMHMM program [42] (https://services.healthtech.dtu.dk/services/TMHMM-2.0/, accessed on 7 October 2025). The ClusterW module of MEGA 12 [43] software was used for multiple-species protein sequence alignment. Based on the alignment results, a phylogenetic tree was constructed using the maximum likelihood method and subjected to bootstrap testing (1000 replicates). Finally, the evolutionary tree was visually optimized using the online tool iTOL [44] (https://itol.embl.de, accessed on 27 October 2025).

### 2.5. Analysis of SPDS Gene Structure and Chromosomal Localization

Homology modeling of the protein’s three-dimensional structure was conducted using the SWISS-MODEL [45] (https://swissmodel.expasy.org, accessed on 7 June 2025) automated protein structure prediction server (Supplementary Figure S2). The coding region sequence (CDS) of the tomato *SPDS* genes and their corresponding genome sequence were obtained. The intron/exon structures of the genes were resolved by sequence alignment using the GSDS online tool [38] (Supplementary Figure S3). The chromosome localization mapping was visualized using TBtools-II software (Supplementary Figure S4).

### 2.6. Identification of Cis-Acting Elements in Promoter Sequence Analysis

A promoter sequence 1500 bp upstream of the start codon of the tomato *SlSPDS* genes was extracted. The sequence was submitted to the PlantCARE database [46] (https://bioinformatics.psb.ugent.be/webtools/plantcare/html, accessed on 15 October 2025) for prediction and annotation of cis-acting elements within the promoter region. Visualization of the prediction results was mapped through the TBtools-II website.

### 2.7. Subcellular Localization of SlSPDS

To determine the subcellular localization of *SlSPDS1-4*. The *SlSPDS1-4* genes were individually cloned from tomato leaf cDNA. Using restriction enzymes SalI/BamHI, they were cloned into the multiple cloning site of the p1300-GFP vector. The constructed recombinant vector was transformed into Agrobacterium tumefaciens strain LBA4404, which was used to transiently infect tobacco leaves via *Agrobacterium*-mediated transformation. The bacterial culture was adjusted to an OD_600_ of 0.6 using MS liquid medium. Five-week-old tobacco plants were selected, and Agrobacterium-mediated transformation was performed by injecting the bacterial suspension onto the abaxial surface of tobacco leaves for transient transformation. Finally, fluorescence signals of the fusion protein (SlSPDS-GFP) were observed using laser confocal microscopy to determine its subcellular localization.

### 2.8. Abiotic Stress Treatment of Tomatoes and Sample Collection

To analyze the expression patterns of target genes, WT potted tomato plants at the five-leaf-heart stage (4 weeks old) were selected and subjected to abiotic stress and hormone treatments under normal watering conditions. All treatment groups included three biological replicates. The fourth fully expanded true leaf samples were collected at 0, 1, 3, 6,9, 12, 24, and 48 h post-treatment. Samples were rapidly frozen in liquid nitrogen and stored at −80 °C for subsequent analysis. Specific treatments were as follows. Temperature stress: Expose potted seedlings to low temperature (4 °C) and high temperature (42 °C) conditions. Salt stress: Irrigated with 100 mL of 300 mM NaCl solution per pot. Drought stress (PEG): Irrigated with 100 mL of 20% PEG6000 solution per pot; the fourth fully expanded true leaf was collected. Abscisic acid (ABA) treatment: Foliar sprayed with 100 μmol/L ABA solution.

OE-*SlSPDS* transgenic lines and WT potted tomato plants at the five-leaf-one-heart stage were subjected to salt and drought stress treatments. All treatments included three biological replicates, with plants maintained under normal watering conditions prior to treatment. Specific treatment and sampling protocols were as follows: Salt stress: Irrigate each pot of overexpressing plants with 100 mL of 350 mM NaCl solution, followed by replenishment with an equal volume of the same solution on day 4 post-treatment. After 7 days of stress, collect the fourth true leaf for DAB staining analysis. Photograph the plants after 10 days of stress treatment to document their overall phenotype. Drought stress (PEG): Each pot was irrigated with 100 mL of 20% PEG solution. The fourth true leaf was collected 3 days after stress onset for DAB staining analysis. Photograph the plants after 10 days of stress treatment to document their overall phenotype. Chlorophyll Content Determination: Fourth to fifth functional leaves were harvested from untreated OE-*SlSPDS1-4* and WT plants. Three biological replicates were established, each comprising four leaves. These leaves were placed in a 200 mM NaCl solution for detached phenotypic observation and chlorophyll content measurement. Results were tabulated as the average values for each genotype.

### 2.9. Detection of Polyamine Content in SlSPDS1-4 Transient Overexpressed Tomato Leaves

To elucidate the regulatory role of the *SlSPDS1-4* in tomato spermidine synthesis, *SlSPDS1-4* overexpression vectors and pG1300 empty vectors were constructed and subsequently transformed into Agrobacterium tumefaciens LBA4404 strain. The bacterial culture was initially activated to an OD_600_ of 0.4–0.5, then further concentrated to an OD_600_ of 0.8–1.0. It was injected into the fourth fully expanded leaf of tomato plants, with at least three biological replicates performed. At 72 h post-injection, the injected leaves were collected, rapidly frozen in liquid nitrogen, and preserved at −80 °C. Polyamine quantification was performed by SanShu Biotechnology (Shanghai, China) using ultra-high-performance liquid chromatography (UPLC).

### 2.10. RNA Extraction, cDNA Synthesis, and qRT-PCR Analysis

Total RNA was extracted using the Vazyme Plant RNA Extraction Kit (Vazyme, Nanjing, China) and verified for purity and integrity via 1.0% agarose gel electrophoresis. cDNA synthesis was performed using the FastKing Reverse Transcription Kit (KR116, Taigen Bio, Beijing, China). Three biological replicates with three technical replicates for each sample were performed. The qRT-PCR reaction system (10 μL) contained 0.5 μL cDNA template, 5 μL TB Green Master Mix (Takara, Beijing, China), and 0.4 pmol of each primer. The qRT-PCR cycling program consisted of an initial denaturation at 95 °C for 30 s, followed by 40 cycles of denaturation at 95 °C for 5 s and annealing/extension at 60 °C for 30 s. The EF1α gene served as an internal control for normalizing cDNA quantity, and relative expression levels of target genes were calculated using the 2^−ΔΔCt^ method.

### 2.11. Determination of Chlorophyll Content in Tomato Leaves and DAB Staining Detection

Chlorophyll content was measured using an SPAD-502 chlorophyll meter (Konica Minolta, Tokyo, Japan). Each sample was measured three times, and the average value was calculated to reduce experimental error. The DAB staining method was used to stain whole leaves from WT and *SlSPDS1-4* overexpressing plants subjected to stress treatment, enabling qualitative observation of hydrogen peroxide (H_2_O_2_) accumulation levels. All data underwent statistical analysis using GraphPad Prism 10 software.

### 2.12. Data Analysis

Data processing and statistical analysis were performed using Microsoft Excel and GraphPad Prism 10. Data were expressed as mean ± standard deviation (SD) of three biological replicates. One-way analysis of variance (ANOVA) was used to assess the significant differences between the control and treated samples, with significance levels set at * *p* < 0.05 and ** *p* < 0.01.

## 3. Results

### 3.1. Identification of SlSPDS1-4 Genes and Analysis of Physicochemical Properties

Four *SlSPDS* genes (*SlSPDS1-4*) encoding spermidine synthase proteins were identified in tomato through bioinformatics database searches (Table 1). The predicted protein length varies from 95 to 357 amino acids, while molecular weights range from 34.20 to 39.26 kDa (*SlSPDS1* being the largest and *SlSPDS2* the smallest). The theoretical isoelectric points (pI) of all *SlSPDS* proteins are below 7.0 (4.81–5.61), suggesting that they are acidic in nature. Protein stability analysis indicated that *SlSPDS1* and *SlSPDS4* are stable proteins, while *SlSPDS2* and *SlSPDS3* are unstable (instability index > 40). The aliphatic index values range from 84.62 to 92.44, reflecting good thermostability. Hydrophobicity analysis revealed that *SlSPDS4*, with an average hydrophobicity index greater than 0, is a hydrophobic protein, whereas *SlSPDS1-3* is neutral. Subcellular localization prediction showed that all *SlSPDS* proteins localized in the cytoplasm (Table 1).

### 3.2. Bioinformatics Analysis and Subcellular Localization of the Tomato SlSPDS1-4 Gene

**Figure 1:**
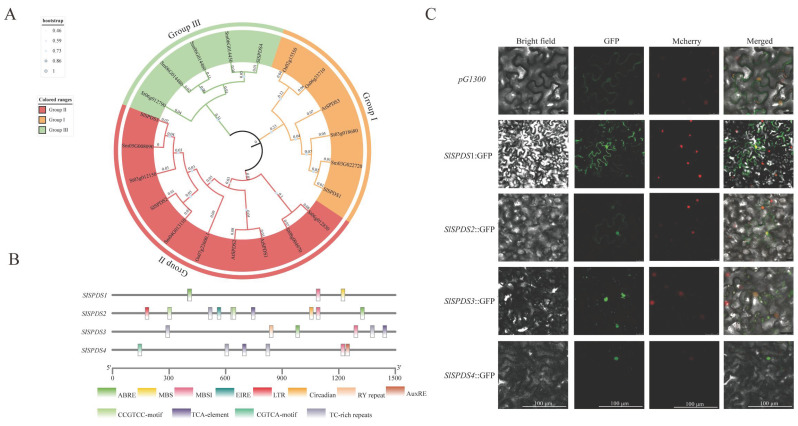
(**A**) The phylogenetic tree is constructed using MEGA 12 software based on multiple sequence alignments, employing the maximum likelihood method with validated bootstrap parameters. Sl: tomato (*S. lycopersicum*); St: potato (*Solanum tuberosum*); Os: rice (*Oryza sativa* L.); Sm: eggplant (*Solanum melongena*); At: *Arabidopsis* (*Arabidopsis thaliana*). (**B**) *SlSPDS* gene promoter cis-acting element prediction. Transcriptional elements were predicted using the PlantCARE database and visualized with TBtools. (**C**) Subcellular localization of the *SlSPDS1-4* genes. Confocal Microscope images showing the subcellular distribution of GFP-tagged S*l*SPDS proteins as well as the control group (pG1300).

To explore the evolutionary relationships of tomato SPDS, a phylogenetic tree was constructed using SPDS protein sequences from five plant species: tomato, potato, rice, *Arabidopsis*, and eggplant (Figure 1A). Based on sequence similarity and evolutionary relationships, phylogenetic analysis classified SPDS proteins into three subgroups (Group I–III): Within Group II, eight sequences clustered together with SlSPDS2 and SlSPDS3 from tomato on a major branch. The evolutionary clustering results indicate that Subgroup III comprises only SPDS sequences from Solanaceae species (tomato, potato, eggplant), representing a Solanaceae-specific SPDS evolutionary lineage. Four SlSPDSs are distributed across three subgroups. Concurrently, tomato SPDS sequences clustered frequently with those from closely related species like potato and eggplant, consistent with the evolutionary principle of closer gene relationships among proximate species. To further explore the potential regulatory mechanisms and stress-related functions of *SlSPDS* genes. A cis-acting element analysis was performed on the 1500 bp promoter regions upstream of the start codons. The analysis revealed a wide distribution of hormone and stress-responsive elements within *SlSPDS* promoters, including abscisic acid-responsive elements, jasmonic acid-responsive motifs, salicylic acid-responsive elements, and auxin-responsive elements. Additionally, elements associated with flavonoid biosynthesis and plant defense were identified. Interestingly, a low temperature-responsive element was exclusively detected in the *SlSPDS2* promoter, while a drought-inducible element was uniquely present in the *SlSPDS1* promoter (Figure 1B). To determine the site where SlSPDS proteins function, subcellular localization analysis was performed (Figure 1C). Recombinant constructs (CaMV 35S::SlSPDS) in the pG1300 vector were transiently expressed in epidermal cells of tobacco leaves. Confocal laser scanning microscopy revealed that SlSPDS1-2 displayed fluorescent signals distributed in both the cytoplasm and nucleus, indicating a nucleocytoplasmic localization pattern similar to that of the pG1300 empty vector control. In contrast, SlSPDS3-4 fluorescence is exclusively detected in the nucleus, confirming its nucleus-specific localization.

### 3.3. Expression Profiles of SlSPDS Genes Under Abiotic Stress Treatments

**Figure 2:**
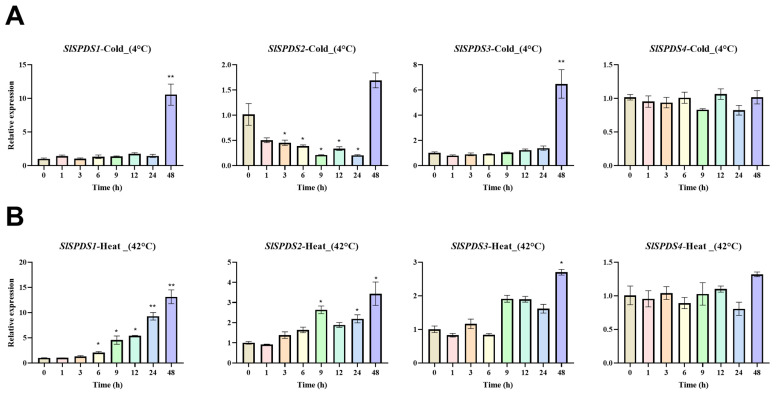
Expression patterns of tomato *SlSPDS1-4* genes under temperature stress: (**A**) high-temperature stress; (**B**) low-temperature stress. The figure displays the temporal expression characteristics of *SlSPDS1-4* genes under two temperature stress conditions. The error bar represents the standard deviation of three biological replicates and three technical replicates. Statistical significance was determined by one-way ANOVA and marked with an asterisk (e.g., * indicates *p* < 0.05, ** indicates *p* < 0.01) when comparing with the 0 h control.

**Figure 3:**
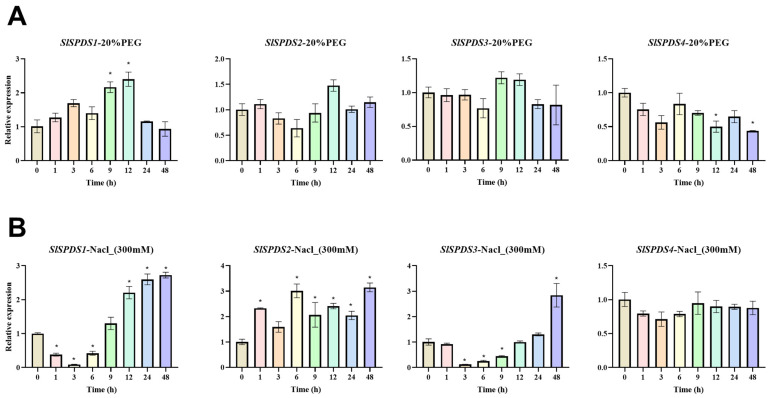
Expression patterns of tomato *SlSPDS1-4* genes under osmotic stress: (**A**) drought stress; (**B**) salt stress. The figure displays the temporal expression characteristics of *SlSPDS1-4* genes under osmotic stress. The error bar represents the standard deviation of three biological replicates and three technical replicates. Statistical significance was determined by one-way ANOVA and marked with an asterisk (e.g., * indicates *p* < 0.05) when comparing with the 0 h control.

**Figure 4:**
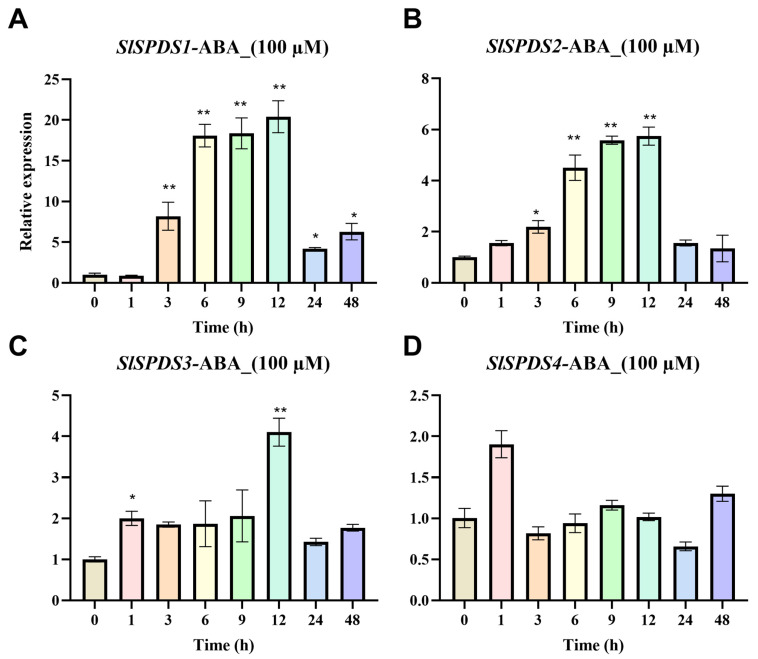
Expression patterns of tomato *SlSPDS1-4* genes under ABA stress: (**A**) *SlSPDS1*, (**B**) *SlSPDS2*, (**C**) *SlSPDS3*, (**D**) *SlSPDS4*. The figure illustrates the temporal expression characteristics of *SlSPDS1-4* genes under ABA stress. The error bar represents the standard deviation of three biological replicates and three technical replicates. Statistical significance was determined by one-way ANOVA and marked with an asterisk (e.g., * indicates *p* < 0.05, ** indicates *p* < 0.01) when comparing with the 0 h control.

To investigate the expression pattern of *SlSPDS1-4* genes under abiotic stress, this study used WT tomato plants as experimental materials, their expression was analyzed by qRT-PCR under temperature stress (4 °C low temperature, 42 °C high temperature) (Figure 2), osmotic stress (300 mM NaCl salt, PEG6000 simulated drought) (Figure 3), and 100 µM ABA treatment (Figure 4).

Under low-temperature stress, the expression patterns of *SlSPDS1-4* genes exhibited significant differences. Among them, *SlSPDS1* and *SlSPDS3* are significantly upregulated at 48 h, with relative expression levels reaching approximately five-fold that of the control group. *SlSPDS2* shows marked downregulation compared to the control, with the downregulation effect being particularly pronounced at 9 h and 24 h. *SlSPDS4* shows no significant fluctuation in expression levels compared to the control group. Under high-temperature conditions, *SlSPDS1* exhibits a gradual increase from 6 h, peaking at 48 h at seven times the control level. *SlSPDS2* gene expression showed an overall upward trend compared to the control group, reaching a significant level at 48 h. The S*l*SPDS3 gene is significantly affected at 48 h. *SlSPDS4* gene expression shows no significant fluctuation compared to the control group (Figure 2).

Under simulated drought stress conditions, compared with the control group, the expression level of the *SlSPDS1* gene is significantly upregulated at 9 h and 12 h post-treatment. *SlSPDS2* gene expression exhibits fluctuating changes, also demonstrating an upward trend at 12 h. *SlSPDS3* gene expression also shows fluctuations, with no significant difference in expression levels compared to the control group during the first 3 h of stress. *SlSPDS4* gene expression exhibits a downregulated trend, with expression levels significantly lower than those of the control group at 48 h. Under the salt stress conditions, *SlSPDS1* gene expression shows a pattern of initial decline followed by an increase compared to the control group, with expression levels significantly higher than the control group, especially in the later stages of stress (after 12 h). The expression level of the *SlSPDS2* gene is generally higher than that of the control group and exhibits fluctuating changes, with expression levels at both the 6 h and 48 h time points significantly higher than those of the control group. The expression of the *SlSPDS3* gene also shows a trend of first decreasing and then increasing, with expression levels at 48 h significantly higher than those of the control group. The expression of the *SlSPDS4* gene shows no significant fluctuations compared to the control group (Figure 3).

Under ABA treatment conditions, *SlSPDS1* gene expression exhibits an upward trend compared to the control group, showing extremely significant upregulation at the 6 h, 9 h, and 12 h time points, with expression peaking at 12 h. *SlSPDS2* gene expression exhibits an initial increase followed by a decrease relative to the control group, also peaking at 12 h with a relative expression level six-fold higher than the control. *SlSPDS3* gene expression remained consistently higher than the control group, reaching a relative expression level four-fold higher than the control at 12 h. *SlSPDS4* gene expression showed an upward trend at 1 h compared to the control group, with no significant fluctuations relative to the control at subsequent time points. Collectively, these results indicate that the *SlSPDS* gene family exhibits differential response patterns to different abiotic stresses, suggesting functional specialization within this gene family (Figure 4).

### 3.4. Impact of SlSPDS Gene Overexpression on Polyamine Metabolism in Tomato Leaves

**Figure 5:**
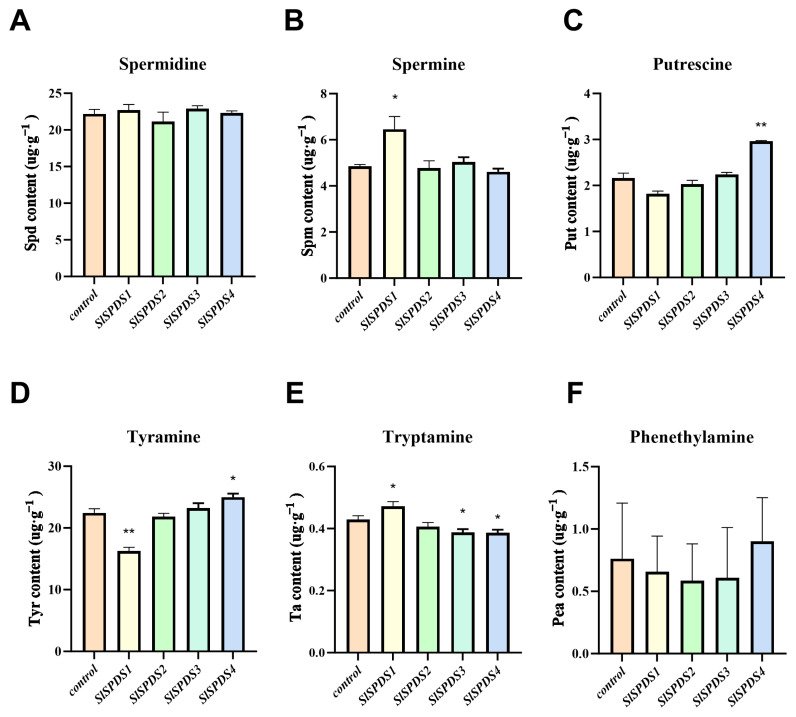
Determination of biogenic amine content in *SlSPDS1-4* gene-overexpressing tomato leaves. The contents of (**A**) spermidine (Spd), (**B**) spermine (Spm), (**C**) putrescine (Put), (**D**) tyramine (Tyr), (**E**) tryptamine (Ta), and (**F**) phenethylamine (Pea) were determined by liquid chromatography. The error bar represents the standard deviation of three biological replicates and three technical replicates. Statistical significance was determined by one-way ANOVA and marked with an asterisk (e.g., * indicates *p* < 0.05, ** indicates *p* < 0.01) when comparing with the control.

To determine the optimal time point for the response of tomato *SlSPDS1-4* genes to spermidine synthesis, we performed transient overexpression of these genes in WT tomato leaves. The results showed that *SlSPDS1* expression is significantly upregulated at 48 h, while *SlSPDS2-4* expression peaked at 72 h (Figure S5). Thus, the polyamine content of 72 h post-injection samples was determined (Figure 5). Results indicate that overexpression of the *SlSPDS1* gene significantly affects the accumulation of spermine, tyramine, and tryptamine in leaves. Overexpression of the *SlSPDS2* gene exerts no statistically significant effect on the content of any polyamine component. Overexpression of *SlSPDS3* significantly affected tryptamine synthesis but exerts no statistically significant effect on other polyamine levels. Overexpression of *SlSPDS4* in tomato plants significantly altered putrescine, tyramine, and tryptamine content but had no significant effect on spermine and spermidine levels. Its expression levels show no statistically significant difference compared to the control group. However, *SlSPDS* genes did not significantly regulate spermidine content.

### 3.5. Phenotypes and DAB Staining Analysis of OE-SlSPDS Transgenic Tomato Seedlings Under 20% PEG6000 Treatment

**Figure 6:**
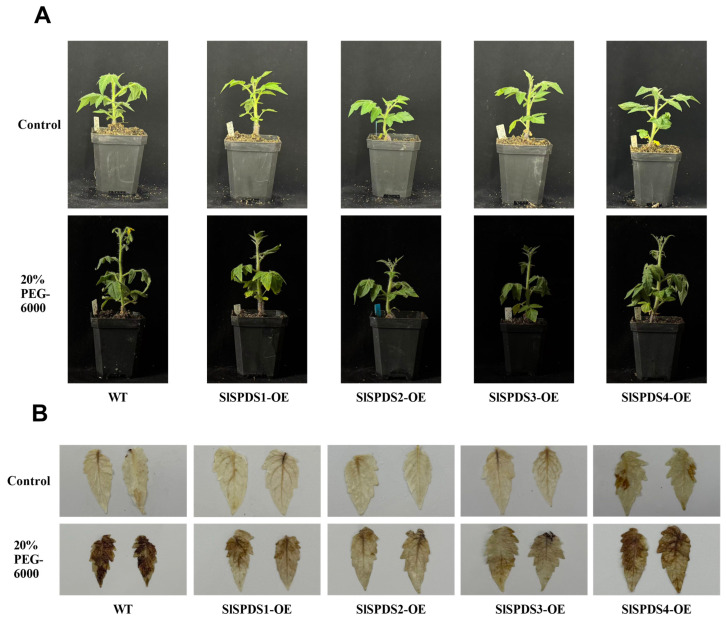
Phenotypes and DAB staining analysis of OE-*SlSPDS* transgenic tomato seedlings under 20% PEG6000 treatment. (**A**) Whole-plant phenotypes of the four-week-old five-leaf-stage OE-*SlSPDS* transgenic tomato seedlings under control conditions and 20% PEG6000 treatment for 10 days. (**B**) DAB staining analysis of the four-week-old five-leaf stage OE-*SlSPDS* transgenic tomato seedlings under 20%PEG treatment. Representative images from three biological replicates are shown.

To elucidate the regulatory role of OE-*SlSPDS* tomato lines in tomato drought tolerance, under drought stress, WT plants showed obvious leaf curling and severe leaf edge scorching, while OE-*SlSPDS* lines displayed slight wilting and better growth performance. We systematically analyzed hydrogen peroxide (H_2_O_2_) accumulation levels in OE-*SlSPDS* tomato leaves under drought stress using DAB staining. The successful overexpression of *SlSPDS* in transgenic tomato lines was confirmed by qRT-PCR analysis (Figure S6). The transcript level of *SlSPDS* in the selected OE line was five-fold higher than that in the WT. These results (Figure 6) revealed that after PEG treatment, WT tomato leaves exhibited strong staining signals ranging from dark brown to blackish brown throughout the tissue, indicating substantial H_2_O_2_ accumulation within the plants. In contrast, DAB staining intensity was reduced in leaves of *SlSPDS* overexpression lines (*SlSPDS1-4*), with distinct phenotypic variations among lines. These results suggest that *SlSPDS*s contribute to the improvement of ROS-scavenging ability in tomato leaves under PEG-induced stress.

### 3.6. Phenotype, DAB Staining, and Chlorophyll Content in OE-SlSPDS Lines Under Salt Stress

**Figure 7:**
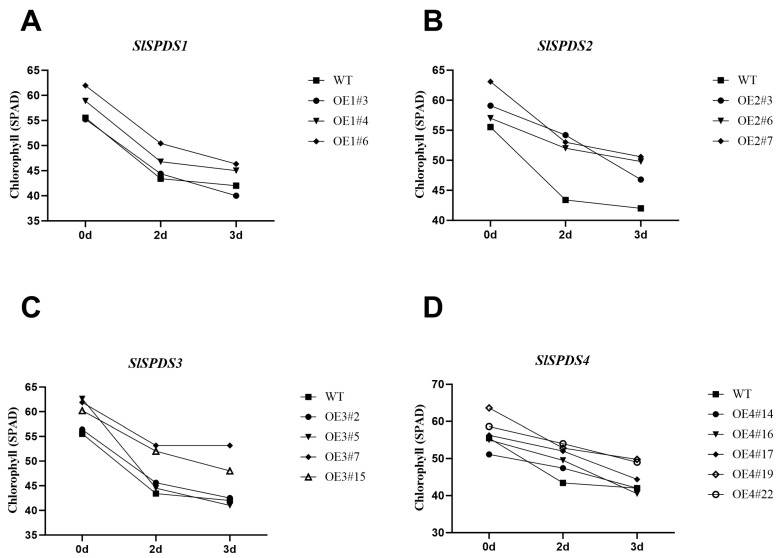
Changes in relative chlorophyll content in the fourth-to-fifth functional leaves of four-week-old, five-leaf-stage OE-*SlSPDS1-4* transgenic and WT tomato plants under detached treatment with 200 mM NaCl. (**A**) OE-*SlSPDS1* (**B**) OE-*SlSPDS2* (**C**) OE-*SlSPDS3* (**D**) OE-*SlSPDS4*. Data are presented as the mean ± standard deviation (SD) of three biological replicates with four leaves per replicate.

**Figure 8:**
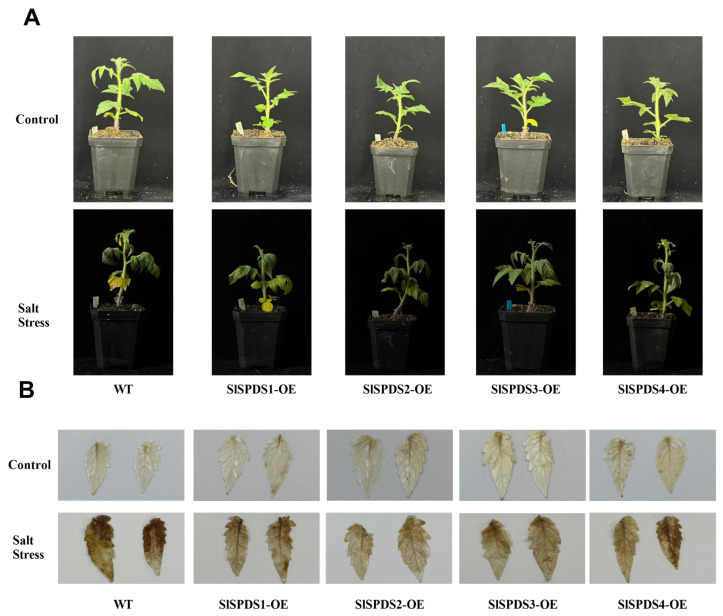
Phenotypes and DAB staining analysis of OE-*SlSPDS* transgenic tomato seedlings under 350 mM NaCl treatment. (**A**) Whole-plant phenotypes of the four-week-old five-leaf-stage OE-S*l*SPDS transgenic tomato seedlings under control conditions and 350 mM NaCl treatment for 10 days. (**B**) DAB staining analysis of H_2_O_2_ accumulation in leaves of four-week-old, five-leaf stage tomato seedlings (OE-*SlSPDS* transgenic lines) under 350 mM NaCl stress. Three independent biological replicates were conducted for each target gene, and representative images are presented.

To investigate the regulatory role of *SlSPDS* genes in tomato salt stress tolerance, under salt stress, WT plants showed serious wilting, significant leaf yellowing, and leaf tip necrosis, whereas OE-*SlSPDS* lines exhibited milder stress symptoms and better growth status. Excised leaves from OE-*SlSPDS* lines were used as materials and subjected to treatment in a 200 mM NaCl solution. Chlorophyll relative content was measured at 0, 2, and 3 days. At the onset of salt stress (0 d), the relative chlorophyll content in WT leaves was lower than that in all OE lines. As stress persisted for 2 and 3 d, chlorophyll content in WT exhibited a sustained and maximal decline, whereas *SlSPDS1-4* overexpressing lines maintained relatively stable chlorophyll levels, with OE-*SlSPDS2* showing the smallest decrease (Figure 7). These results indicate that *SlSPDS* gene overexpression enhances chlorophyll stability in tomato leaves under salt stress. DAB staining under 350 mM NaCl stress conditions revealed that (Figure 8) leaves of the WT exhibited extensive continuous dark brown precipitates, indicating massive hydrogen peroxide bursts within cells, whereas oxidative stress was significantly alleviated in OE-*SlSPDS1-4*. OE-*SlSPDS2* plants exhibited mild overall leaf discoloration, while OE-*SlSPDS1*, OE-*SlSPDS3*, and OE-*SlSPDS4* plants displayed only localized pale brown spots.

## 4. Discussion

Polyamines are key regulatory factors in plant growth and development. SPDS, as the core enzyme in the polyamine biosynthesis pathway, maintains polyamine homeostasis and participates in the regulation of growth and development [47,48]. Research has confirmed that polyamines not only directly regulate developmental processes as plant growth regulators but also act as second messengers for plant hormones, focusing on stress resistance in response to transpiration stress and synergistically controlling plant growth and development [49,50]. In this study, we first analyzed the gene structures and conserved motifs of *SlSPDS* family members to clarify their basic characteristics. Subsequently, subcellular localization was performed to determine the site of protein function. Finally, expression patterns under abiotic stresses were detected to reveal their potential involvement in stress tolerance. These results systematically lay a foundation for exploring the biological functions of S*l*SPDS genes in tomato. This finding is consistent with previous studies demonstrating AtPAO2 involvement in spermidine-mediated seed germination and early morphogenesis [51]. The regulatory role of polyamines covers the entire process of plant growth and development, participating in key developmental stages such as flowering, fruit ripening, and senescence [52,53].

Salt and drought stresses jointly inhibit plant growth by inducing physiological disorders such as ion imbalance, oxidative damage, cellular dehydration, and metabolic disruption [54]. Previous studies have demonstrated that spermidine significantly mitigates the adverse effects of salt stress by regulating nutrient metabolism in tomato leaves, enhancing sodium ion efflux capacity, and mediating SOS1-dependent long-distance sodium ion transport [55,56,57]. Under salt stress, *SlSPDS1* and *SlSPDS3* exhibited fluctuating induced expression, while *SlSPDS2* maintained significantly higher expression levels than the control throughout the stress period. Under PEG6000-simulated drought stress, *SlSPDS1* exhibited the most pronounced response, while other family members showed weaker responses, indicating this gene has high specificity and sensitivity to drought stress. Phenotypic observations showed that OE-*SlSPDS* significantly enhanced drought and salt tolerance in tomato. Under drought stress, WT plants exhibited severe leaf curling, while OE-*SPDS* lines grew better. Under salt stress, WT plants displayed obvious wilting, leaf yellowing, and tip necrosis, whereas OE-*SPDS* lines showed milder symptoms. These results demonstrate that OE-*SlSPDS* improves tomato tolerance to drought and salt stress. Previous studies have demonstrated the relationship between polyamines and plant growth and development under drought conditions in wheat, indicating that spermine and spermidine can alleviate the adverse effects of drought on plants [58]. Furthermore, Capell et al. confirmed that transgenic rice plants generated by regulating the polyamine biosynthesis pathway exhibited significantly elevated polyamine levels and consequently enhanced drought tolerance [59]. Salt stress and drought stress induce bursts of reactive oxygen species (ROS) in plants. Excessive ROS disrupts cell membrane integrity and damages organelles, serving as a key factor leading to stress-induced damage in plants [60,61]. The results showed that WT tomato leaves exhibited deep brown DAB staining after stress conditions, whereas OE-*SlSPDS* lines showed reduced staining intensity with localized spots or mild staining. These observations indicate that overexpression of *SlSPDS* inhibits H_2_O_2_ accumulation in tomato leaves under abiotic stress, enhances the ROS-scavenging capacity of plants, and thereby alleviates stress-induced oxidative damage. Chlorophyll, the central pigment in photosynthesis, is disrupted by abiotic stress through impaired synthesis pathways and accelerated degradation, leading to leaf yellowing and reduced photosynthetic efficiency. Chlorophyll content serves as a crucial physiological indicator for evaluating plant stress tolerance [62]. Notably, the OE-*SlSPDS* lines exhibited higher initial chlorophyll content under non-stress conditions, indicating that the excessive production of spermidine enhances the basal physiological state of tomato leaves. This elevated chlorophyll level likely provides crucial physiological support for the improved stress tolerance of OE lines. The rate of chlorophyll decline in the overexpressing lines was lower than that in the WT, indicating that OE-*SlSPDS* mitigates the damage to tomato chlorophyll caused by abiotic stress. In addition to osmotic stress, temperature stress significantly disrupts membrane stability in tomato cells and inhibits enzyme activity, thereby affecting normal plant growth and development [63]. This study found that *SlSPDS1-3* genes were significantly upregulated under both high and low temperature stress, reaching peak expression at 48 h after treatment. Notably, the early activation of *SlSPDS1–3* under low-temperature stress suggests that their expression may be modulated by low-temperature response elements (LTRs). As a central hub in abiotic stress signaling, ABA mediates adaptive responses to various stresses, including osmotic and thermal stress in plants [64]. Under treatment with 100 µM ABA, *SlSPDS1-3* were significantly upregulated at all examined time points, whereas *SlSPDS4* showed no significant difference relative to the control. These results indicate that this gene family displays differential responsiveness to ABA signaling. Previous studies have demonstrated a positive correlation between *SPDS* expression and endogenous ABA content [11]. Conversely, in ABA-deficient plants, *SPDS* transcript levels fail to increase significantly even under stress conditions, implying that ABA exerts a conserved transcriptional regulatory effect on *SPDS* genes [49]. Transformation of the apple *SPDS* gene into European pear significantly increases endogenous spermidine content in plants and enhances tolerance to multiple stresses, confirming the functional conservation of this gene in plant stress response regulation [65]. The *SlSPDS* gene family’s differential responses to various abiotic stresses clearly demonstrate its functional specialization. Although the expression of *SlSPDS4* was weakly induced under stress, it possessed a conserved sequence and a typical *SPDS* domain. To fully reveal the function of the tomato *SlSPDS* gene family, we still selected it for overexpression analysis. The results showed that overexpression of *SlSPDS4* also improved stress tolerance in tomato.

Previous studies have demonstrated that elevated expression of *SPDS* genes in other plants significantly regulates the anabolic metabolism of Spd and other polyamines. For instance, overexpression of *GhSAMDC1* enhances salt tolerance in Arabidopsis, with the mechanism likely involving promotion of the polyamine synthesis pathway and regulation of Put/Spd/Spm levels [66]. In rice, activation of the *OsSPDS* genes also effectively promotes Spd accumulation, thereby influencing the plant’s stress resistance [67]. In tobacco, silencing the *NtSPDS* gene results in reduced Spd content, while its precursor Put and downstream product Spm show slight increases [25]. Overexpression of the *MdSPDS1* gene in sweet orange (*Citrus sinensis* Osbeck) significantly enhances stress resistance [68]. This study employed *Agrobacterium*-mediated transient transformation technology to overexpress *SlSPDS1-4* genes in tomato leaves. Sampling results indicated that *SlSPDS1* expression was significantly upregulated at 48 h and 72 h, while *SlSPDS2-4* expression levels peaked at 72 h (Supplementary Figure S5). Thus, we standardized the sampling time to 72 h. Despite the substantial increase in transcriptional levels across all genes, their overexpression did not significantly affect leaf spermidine content. This outcome may be closely related to the characteristics of the transient expression system: the polyamine synthetic metabolic network exhibits high complexity, involving multiple steps such as amino acid precursor supply and intermediate metabolite conversion. Expressed exogenous genes in transient expression systems function only for a brief period. The 72 h expression window may be insufficient to drive the complete metabolic flux redirection of the spermidine synthesis pathway, thereby failing to produce detectable, significant differences in spermine accumulation. Extending the sampling period might reveal noticeable changes in spermidine levels. Notably, although *SlSPDS* genes exhibit limited regulatory effects on spermidine, they demonstrate specific control over the synthesis of other polyamines and related aromatic amines. Based on phenotypic observations and the regulatory role of *SlSPDS* in spermidine synthesis, these results indicate that *SlSPDS* overexpression promotes spermidine accumulation, thereby enhancing ROS scavenging capacity, suppressing H_2_O_2_ accumulation, reducing oxidative damage and membrane lipid peroxidation, and ultimately improving stress resistance. And these results conclusively demonstrate that plant polyamine synthesis constitutes an interconnected, complex metabolic network. Expression changes in individual polyamine synthesis-related genes can significantly regulate the synthesis of other polyamines through cascading effects within the metabolic network [69,70]. Compared to other species, *SPDS* genes exhibit relatively uniform response patterns to abiotic stress, and most related studies focus on single-function validation rather than systematic analysis. For instance, tobacco *NtSPDS* demonstrates only a single effect, decreased Spd content following gene silencing [25], while *OsSPDS* merely activates under stress to promote Spd accumulation and enhance drought tolerance, without functional differentiation among family members [67]; in wheat, only the mitigating effect of Spd/Spm against drought injury has been validated [58]; only the enhancement of multiple stress tolerances by *MdSPDS1* overexpression has been demonstrated, without detailed physiological parameter analysis [68]. In contrast, the four *SlSPDS* genes in tomatoes exhibited distinct differential expression patterns in response to abiotic stress in this study, with *SlSPDS4* showing particularly weak responsiveness to abiotic stress.

## 5. Conclusions

In summary, four spermidine synthase genes (*SlSPDS1–SlSPDS4*) were identified in tomato, encoding mainly stable acidic proteins localized in the cytoplasm and nucleus. Phylogenetic and promoter analyses revealed high evolutionary conservation and multiple hormone and stress-responsive elements, suggesting regulatory roles in stress adaptation. Overexpression experiments confirmed that *SlSPDS* genes influence polyamine accumulation and serve as a potential target for enhancing tomato stress tolerance. Using tomato lines overexpressing the *SlSPDS* genes, this study investigated the gene’s function in tomato responses to abiotic stresses (drought and salt stresses) through DAB staining and chlorophyll content measurements. Combining stress phenotypes with physiological indicator results suggests that *SlSPDS* overexpression may enhance tomato tolerance to drought and salt stress by boosting ROS scavenging capacity and mitigating stress-induced chlorophyll degradation.

## Figures and Tables

**Table 1:** Analysis of tomato spermidine family members and physicochemical properties.

Gene Name	Gene ID	pI	Molecular Weight (Da)	ORF Length (bp)	Number of Amino Acids (aa)	Instability Index	Aliphatic Index	Grand Average of Hydrophathicity	Amino Acid Length (aa)	Subcellular Localization
*SlSPDS1*	Solyc03g007240.2.1	5.61	39,261.13	1071	356	39.59	88.37	−0.082	357	cytoplasm
*SlSPDS2*	Solyc04g026030.2.1	5.23	34,201.97	930	309	49.64	87.28	−0.121	310	cytoplasm
*SlSPDS3*	Solyc05g005710.2.1	4.81	37,678.90	285	342	56.18	84.62	−0.160	95	cytoplasm
*SlSPDS4*	Solyc06g053520.2.1	5.09	35,098.39	951	316	33.97	92.44	0.034	317	cytoplasm

## Data Availability

The data presented in this study are available in this article or the Supplementary Material.

## Supplementary Materials

The following supporting information can be downloaded at: https://www.mdpi.com/article/10.3390/cells15060533/s1, Figure S1: The protein sequences of the tomato *SlSPDS* gene family were analyzed using the MEME online tool and a total of 7 conserved motifs (Motifs) were identified; Figure S2: The results of protein tertiary structure analysis showed that *SlSPDS2* and *SlSPDS3* have highly similar spatial conformations; Figure S3: Analysis of the gene structure showed that all members of the tomato *SlSPDS* family contain introns and their structures were relatively complex; Figure S4: Chromosomal localization visualization of *SlSPDS* family members was performed using TBtools; Figure S5: Transient expression analysis of *SlSPDS1-4* genes in WT tomato using pG1300 as the empty vector control. Figure S6: Relative expression levels of *SlSPDS* gene in transgenic tomato overexpression (OE) lines (OE-*SlSPDS1-4*) and WT plants verified by quantitative real-time PCR (qPCR).
