# Supplementary material for: Functional Analysis of Tomato SPDS in Response to Osmotic Stress
Source: Cells. 2026 Mar 17;15(6):533. doi: 10.3390/cells15060533 (PMC13025490; doi:10.3390/cells15060533)
Supplement: Supplementary file 1 [file cells-15-00533-s001.zip › Figure S.pdf]

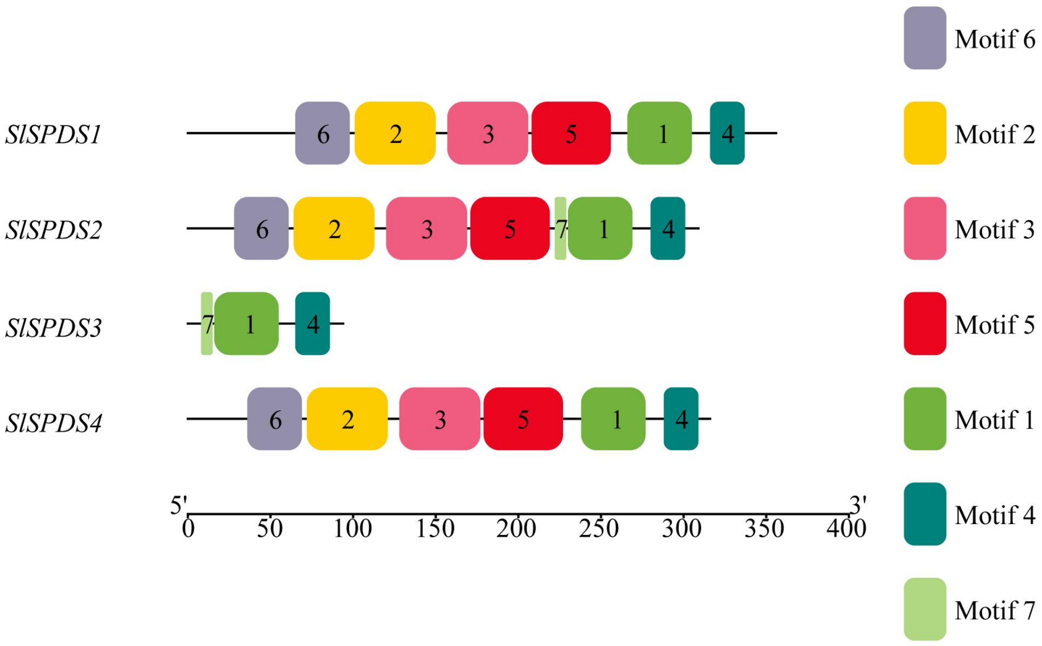

Figure S1. The protein sequences of the tomato *SlSPDS1-4* gene family were analyzed using the MEME online tool, identifying seven conserved motifs. As shown in Table 3, the conserved motifs present in d ifferent SlSPDS family members exhibit variations.

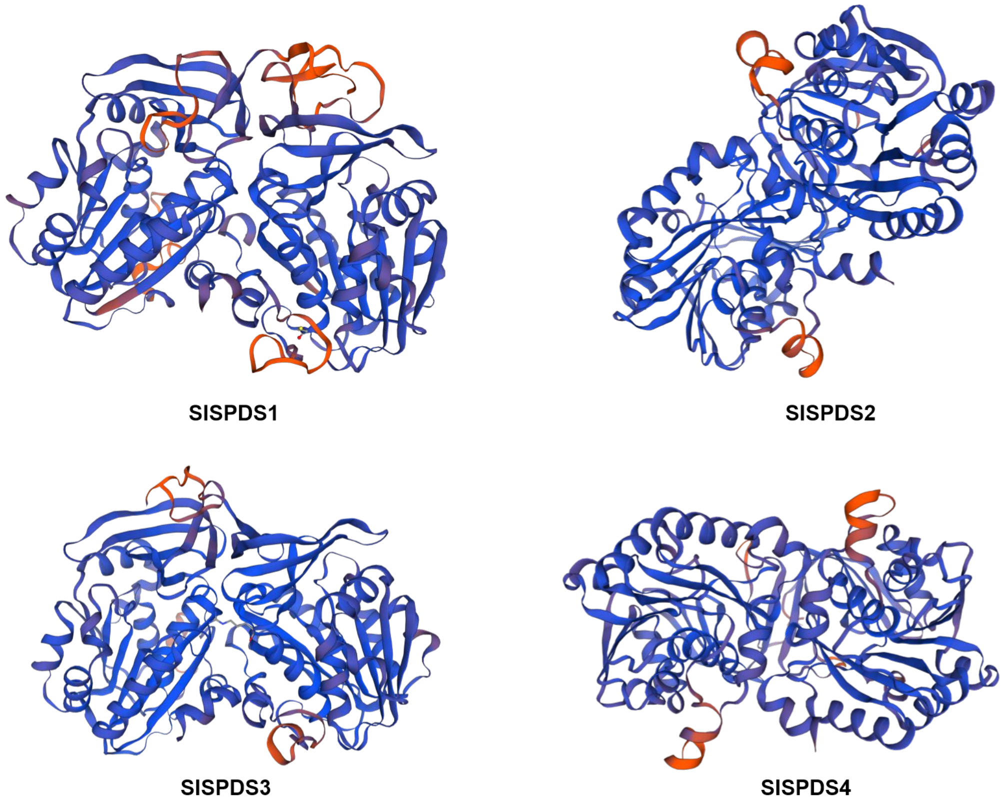

Figure S2. Three-dimensional structural models of the SlSPDS protein family.Homology models of SlSPDS1-4 generated using the SWISS-MODEL platform via homology modeling. In the structural models, the overall main-chain folding architecture of each protein is depicted in blue; orange-highlighted regions indicate key conserved functional domains critical for the enzymatic activity of SlSPDS proteins.

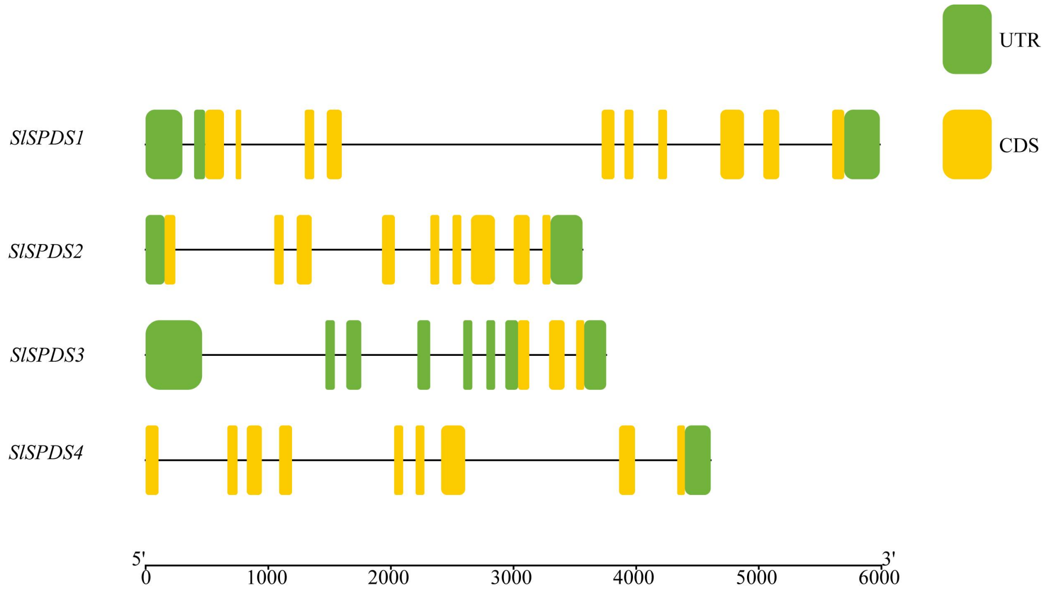

Figure S3. Intron-Exon Structural Framework of the Tomato *SlSPDS* Gene Family.The intron-exon structures of *SlSPDS1-4* were visualized using the online tool GSD. Gene names (*SlSPDS1-4*) are labeled to the left of their corresponding structures.

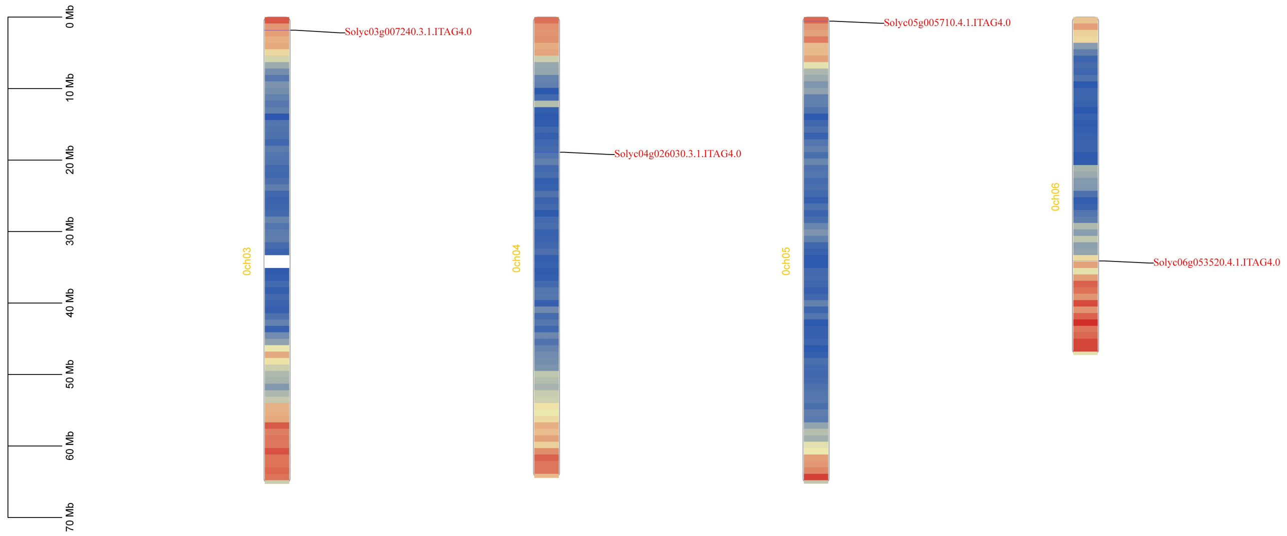

Figure S4. Chromosomal Localization of the *SlSPDS* Gene Family in Tomato.The chromosomal distribution of *SlSPDS1-4* was visualized using TBtools software. Figure legend:*SlSPDS1-4* are located on four distinct chromosomes in tomato, with one *SlSPDS* gene present on each of chr01, chr02, chr03, and chr04.

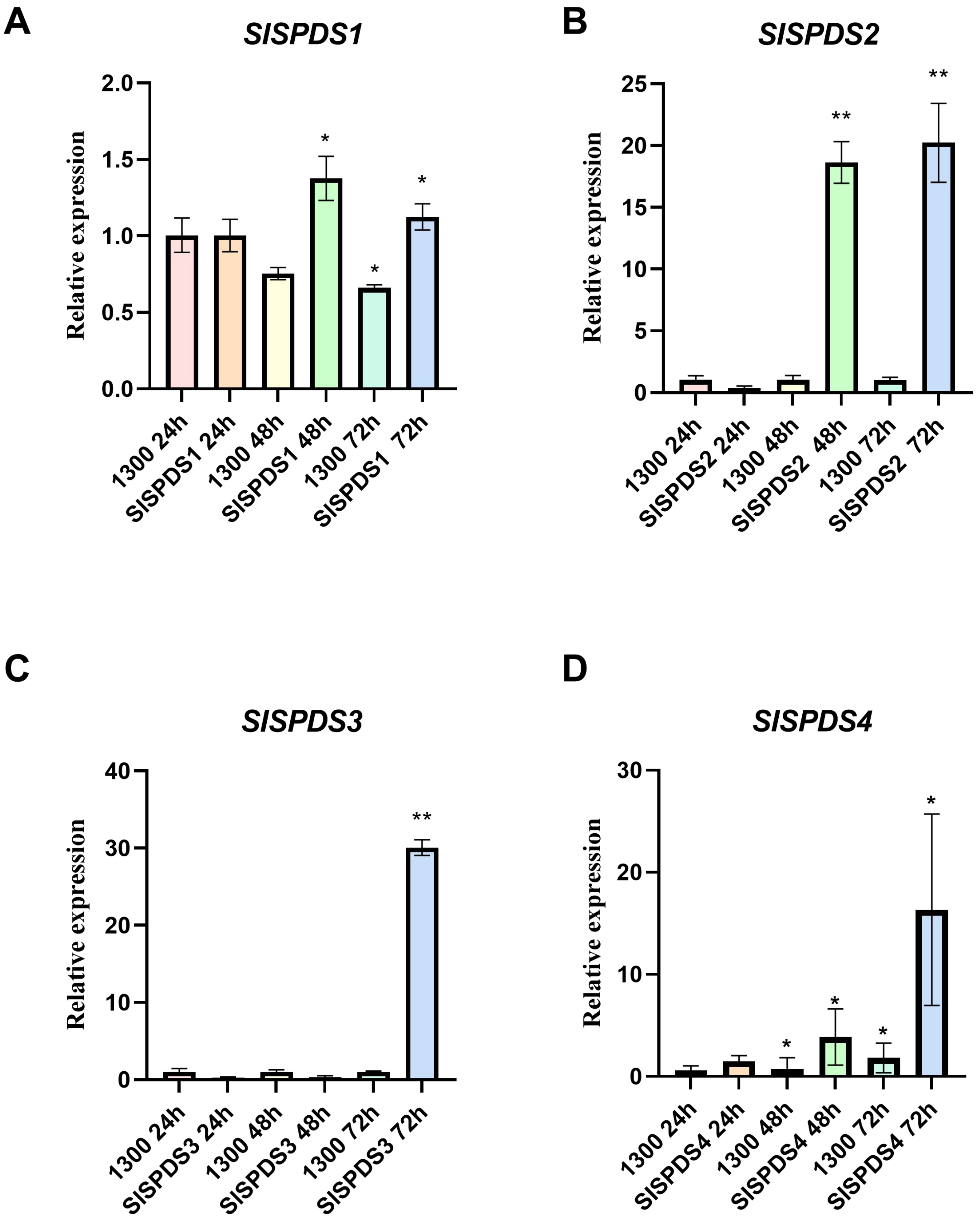

Figure S5. Transient expression analysis of *SlSPDS1-4* genes in WT tomato using pG1300 as the empty vector control. Each bar corresponds to the relative expression level of a specific gene in the transient expression assay. Error bars denote the standard deviation from three biological replicates. Statistical significance is indicated by asterisks (* for $P < 0.05$, ** for $P < 0.01$) when comparing the expression levels of each *SlSPDS* gene with that of the pG1300 empty vector control. These results demonstrate the differential transient expression patterns of the four SlSPDS genes in wild-type tomato.

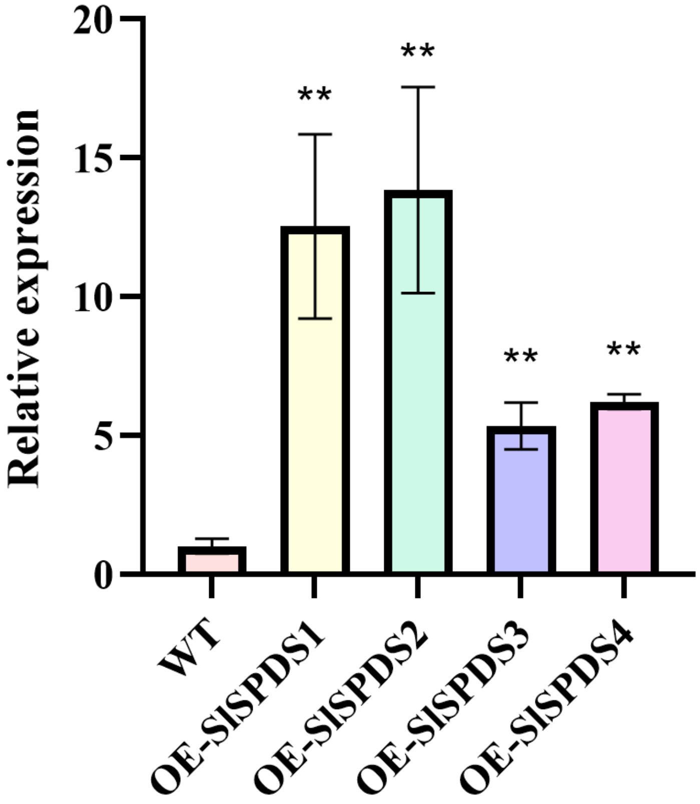

Figure S6. Relative expression levels of SlSPDS gene in transgenic tomato overexpression (OE) lines (OE -*SlSPDS1-4*) and WT plants verified by quantitative real-time PCR (qPCR). Data are presented as mean ± standard deviation (SD) from three biological replicates (each with three technical replicates). Error bars represent the standard deviation of three biological replicates. Statistical significance was determined by one – way ANOVA, where * denotes $p < 0.05$, ** denotes $p < 0.01$.
