# Supplementary figures and images for: Functional Analysis of Tomato SPDS in Response to Osmotic Stress
Source: Cells. 2026 Mar 17;15(6):533. doi: 10.3390/cells15060533 (PMC13025490; doi:10.3390/cells15060533)

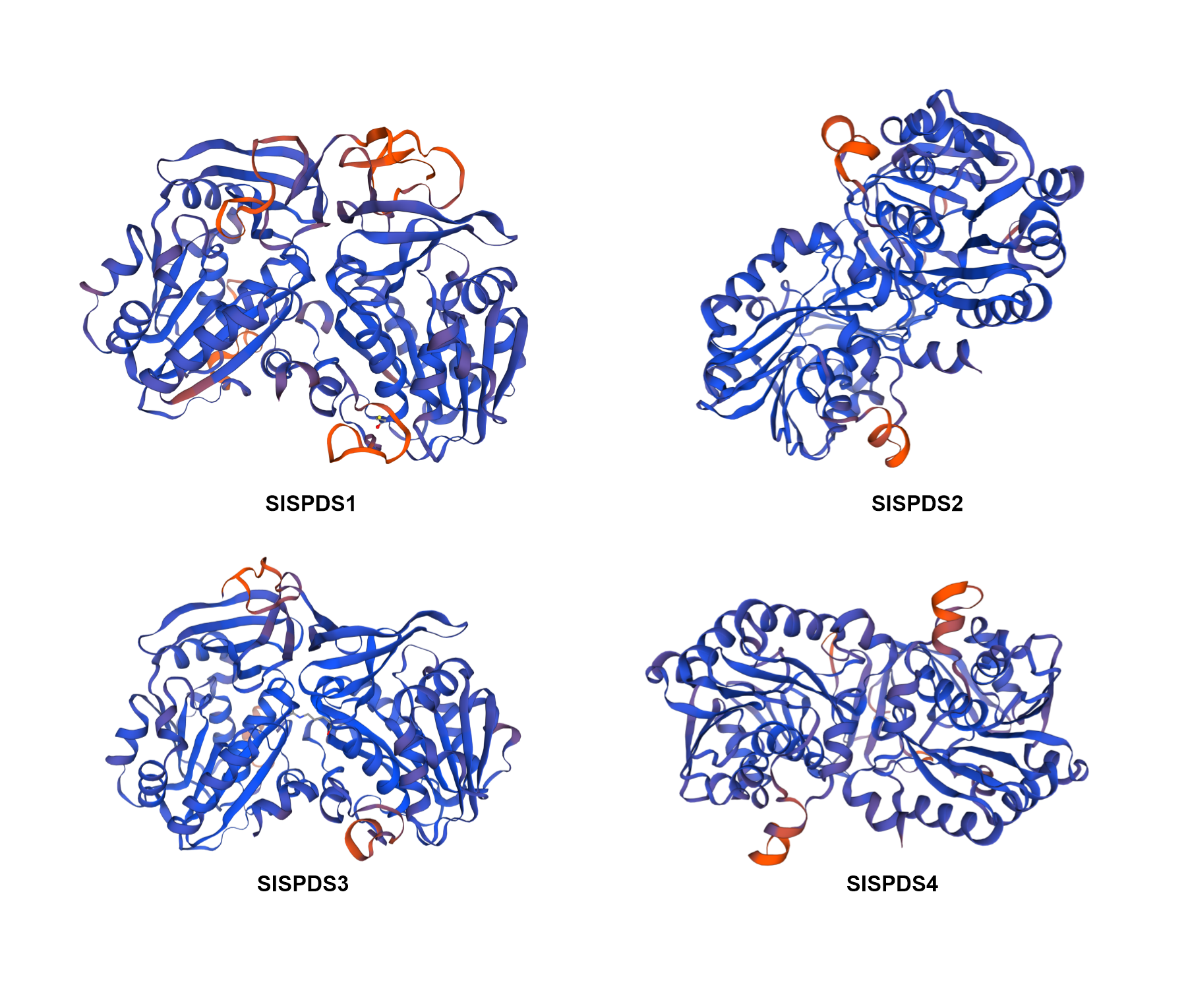

Supplement: Supplementary file 1 [file cells-15-00533-s001.zip › Figure S2.tif]

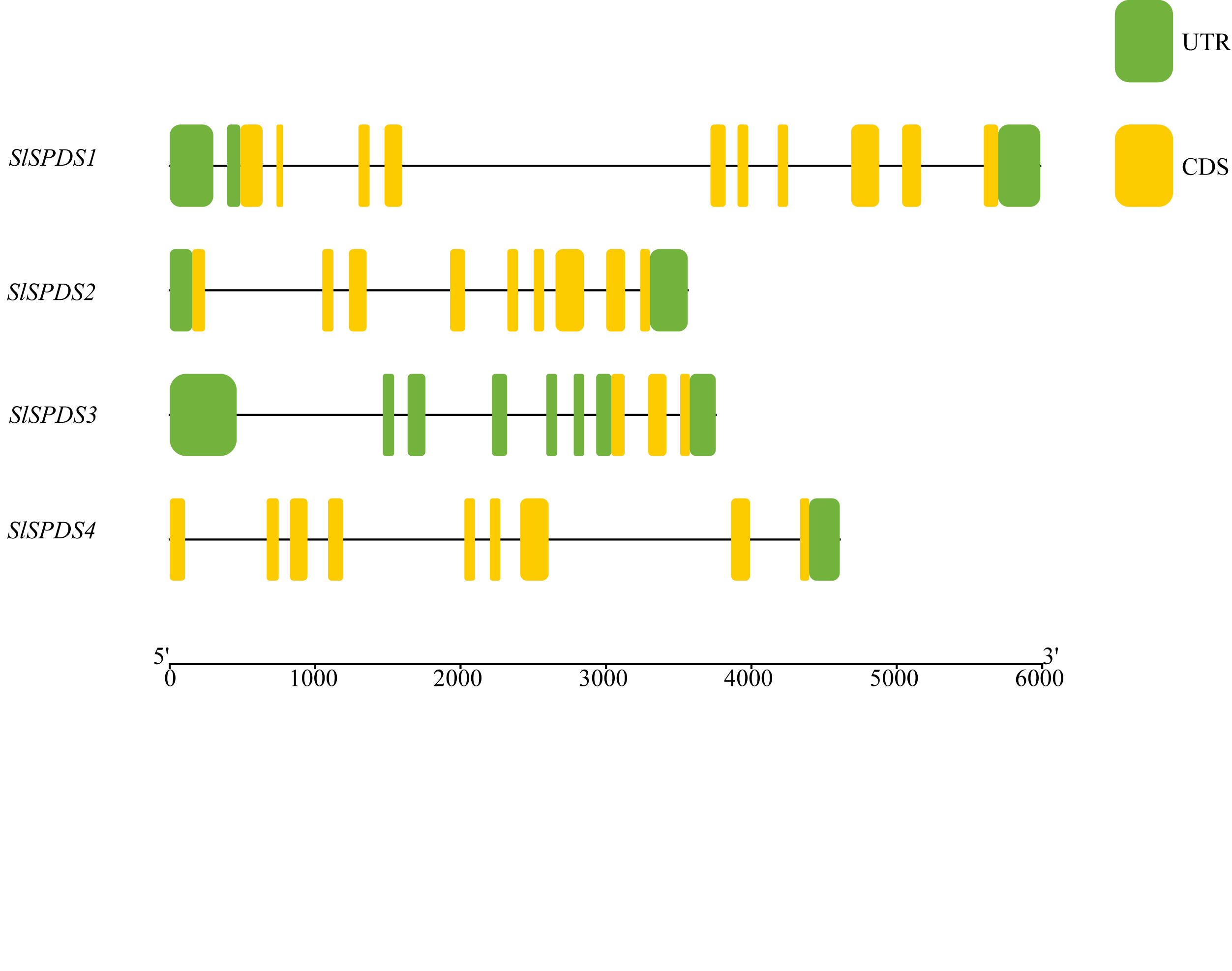

Supplement: Supplementary file 1 [file cells-15-00533-s001.zip › Figure S3.tif]

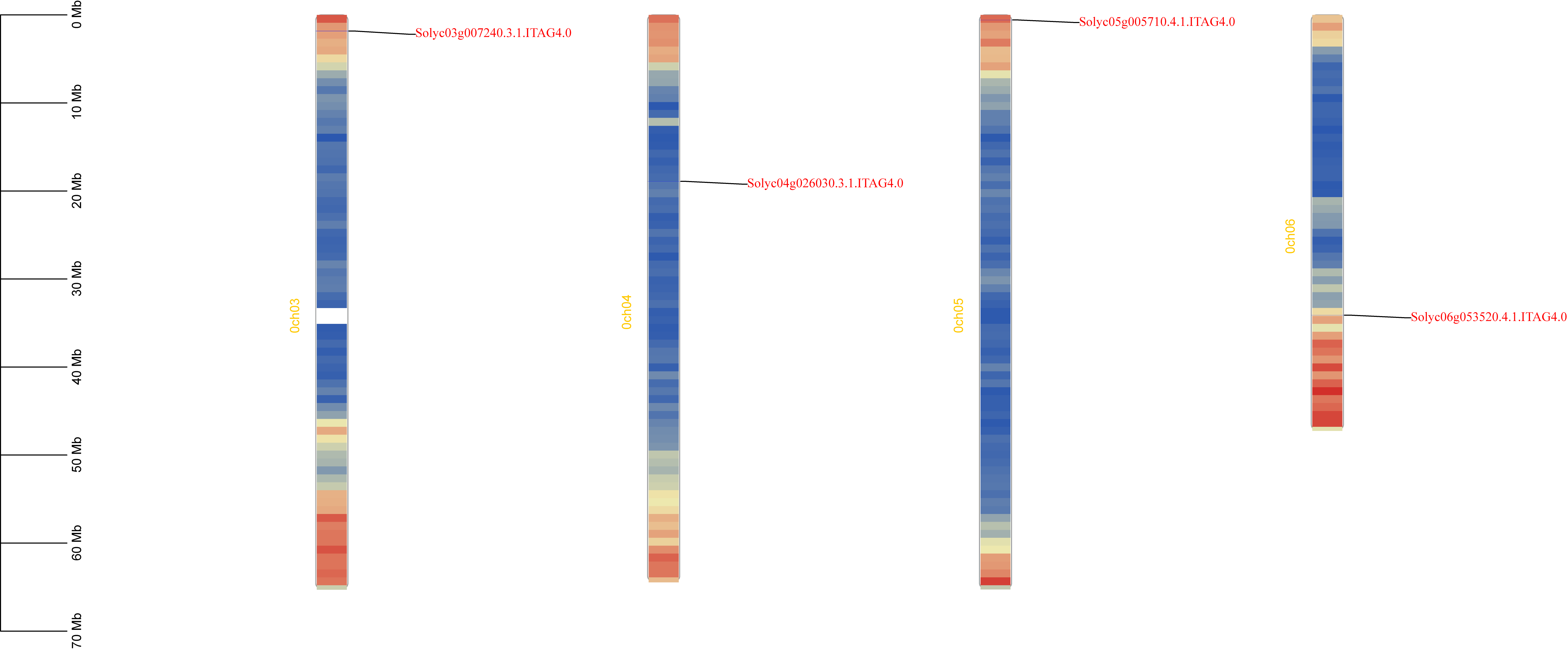

Supplement: Supplementary file 1 [file cells-15-00533-s001.zip › Figure S4.tif]

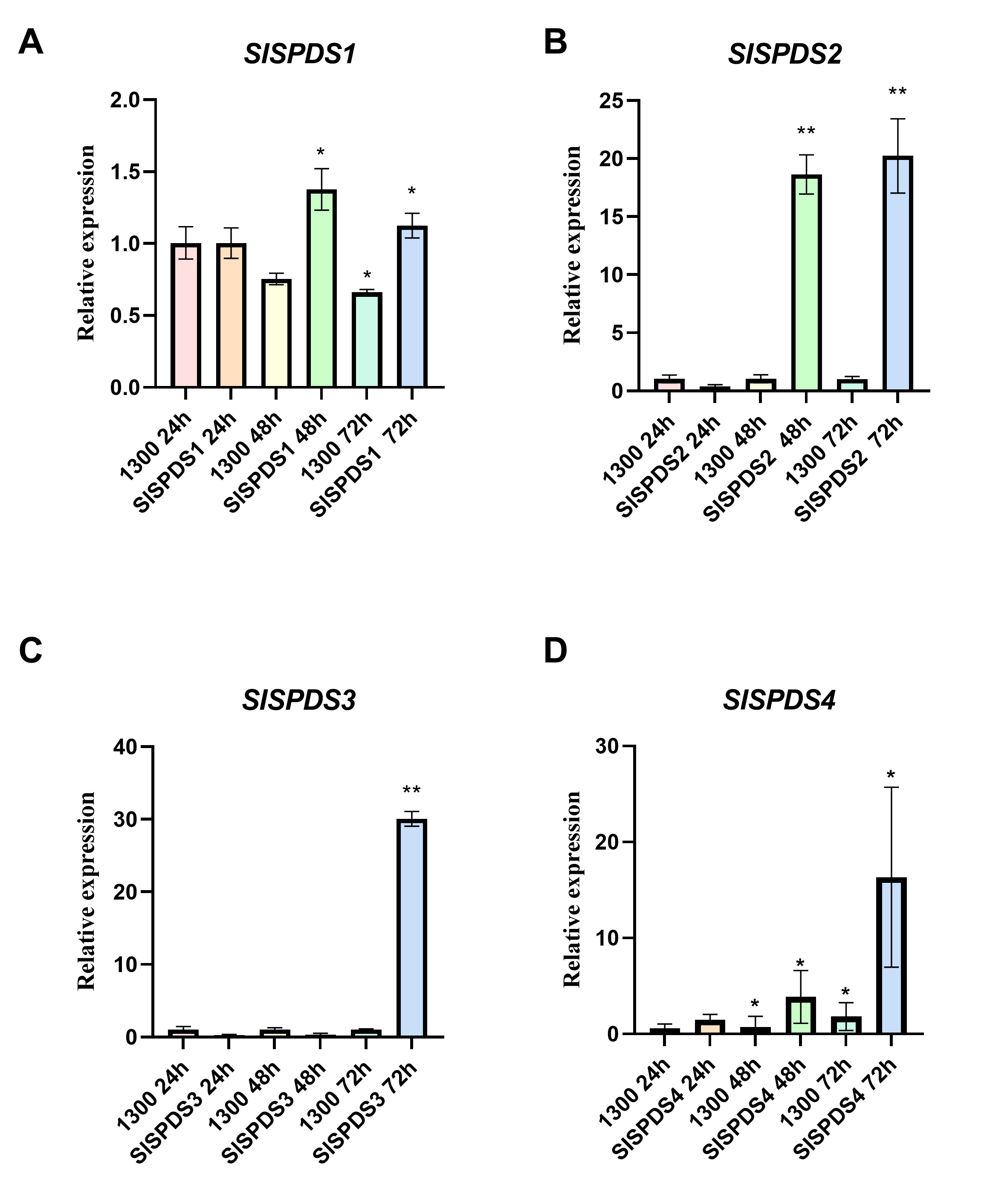

Supplement: Supplementary file 1 [file cells-15-00533-s001.zip › Figure S5.tif]

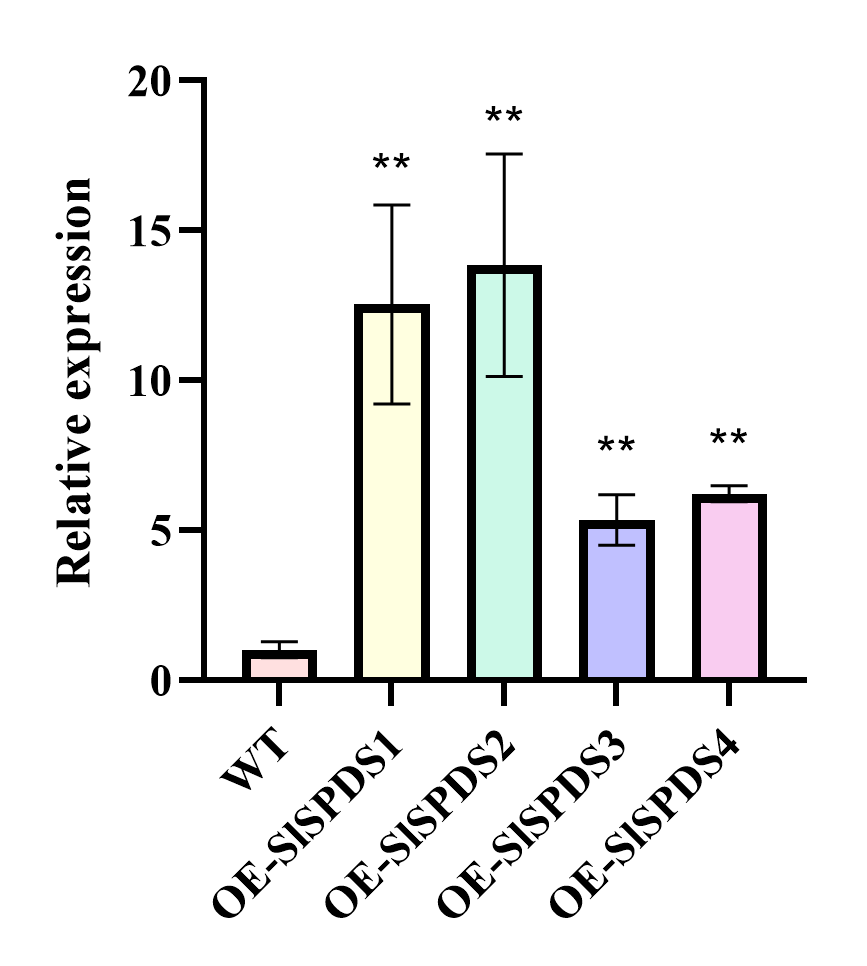

Supplement: Supplementary file 1 [file cells-15-00533-s001.zip › Figure S6.tif]

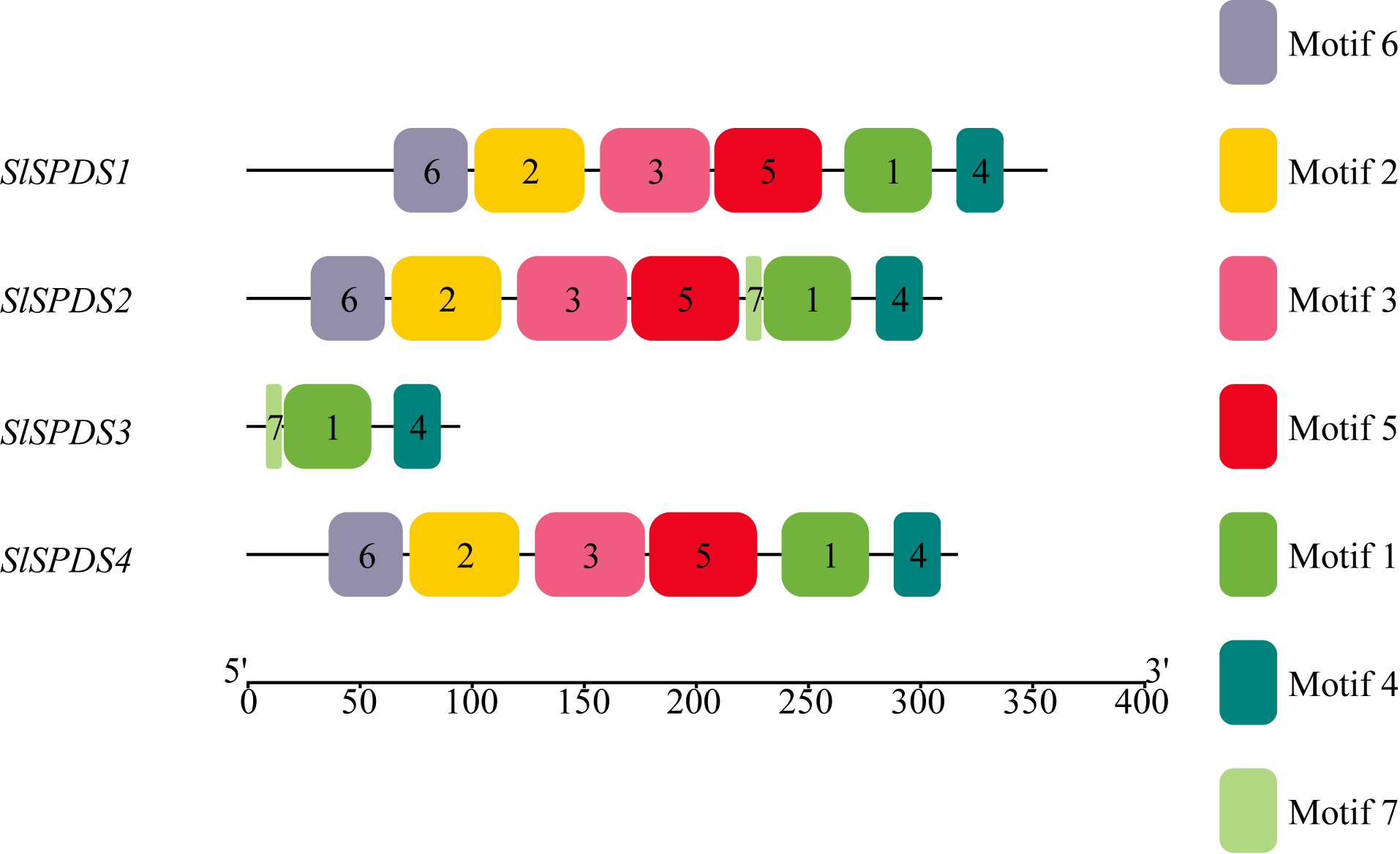

Supplement: Supplementary file 1 [file cells-15-00533-s001.zip › Figure S1.tif]
